# Fractional excretion of total protein predicts renal prognosis in Japanese patients with primary membranous nephropathy

**DOI:** 10.1093/ckj/sfae071

**Published:** 2024-03-20

**Authors:** Hideaki Kuno, Go Kanzaki, Takaya Sasaki, Yusuke Okabayashi, Kotaro Haruhara, Shinya Yokote, Kentaro Koike, Hiroyuki Ueda, Nobuo Tsuboi, Takashi Yokoo

**Affiliations:** Division of Nephrology and Hypertension, The Jikei University School of Medicine, Tokyo, Japan; Division of Nephrology and Hypertension, The Jikei University School of Medicine, Tokyo, Japan; Division of Nephrology and Hypertension, The Jikei University School of Medicine, Tokyo, Japan; Division of Nephrology and Hypertension, The Jikei University School of Medicine, Tokyo, Japan; Division of Nephrology and Hypertension, The Jikei University School of Medicine, Tokyo, Japan; Division of Nephrology and Hypertension, The Jikei University School of Medicine, Tokyo, Japan; Division of Nephrology and Hypertension, The Jikei University School of Medicine, Tokyo, Japan; Division of Nephrology and Hypertension, The Jikei University School of Medicine, Tokyo, Japan; Division of Nephrology and Hypertension, The Jikei University School of Medicine, Tokyo, Japan; Division of Nephrology and Hypertension, The Jikei University School of Medicine, Tokyo, Japan

**Keywords:** clearance, fractional excretion of total protein, nephrotic syndrome, primary membranous nephropathy, renal prognosis

## Abstract

**Background:**

Primary membranous nephropathy (pMN) is one of the most common types of glomerulonephritis, with a third of patients progressing to renal insufficiency. Various prognostic factors have been reported, of which urinary protein and renal function are the most critical parameters. Fractional excretion of total protein (FETP) indicates protein leakage that accounts for creatinine kinetics and serum protein levels. In this study, we investigated the association between FETP and renal prognosis in pMN.

**Methods:**

We retrospectively identified 150 patients with pMN. FETP was calculated as follows: (serum creatinine × urine protein)/(serum protein × urine creatinine) %. We divided the patients into three groups according to FETP values and compared the clinicopathological findings. The primary outcome was an estimated glomerular filtration rate (eGFR) decrease of ≥30% from the baseline level.

**Results:**

FETP was associated with urinary protein and renal function, Ehrenreich and Churg stage, and global glomerulosclerosis. The primary outcome was observed in 38 patients (25.3%), and the frequency of the primary outcome was higher in the high FETP group (*P* = .001). FETP is higher than protein–creatinine ratio (PCR) in the area under the curve. In the multivariate analysis adjusted for age, eGFR, PCR and treatment, FETP was significantly associated with primary outcome (adjusted hazard ratio, 8.19; *P* = .019).

**Conclusions:**

FETP is a valuable indicator that can reflect the pathophysiology and is more useful than PCR as a predictor of renal prognosis in patients with Japanese pMN.

## Graphical Abstract

**Graphical Abstract:**
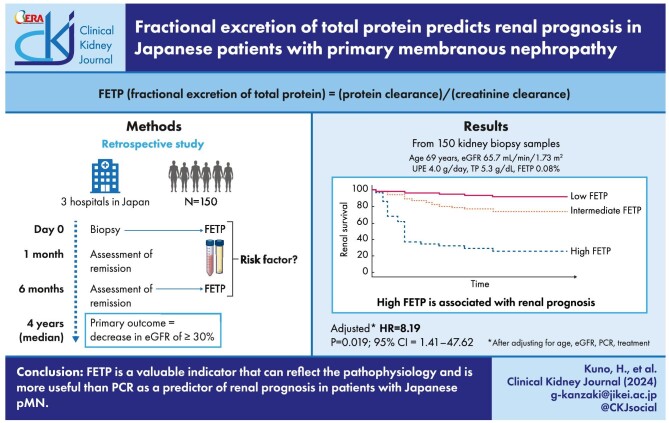

## INTRODUCTION

Primary membranous nephropathy (pMN) is one of the most common types of adult-onset glomerulonephritis conditions [1–3]. In studies ranging 3–5 years, the percentage of patients with pMN that progress to end-stage kidney disease (ESKD) is 5%–9%. Despite improvements in treatment, pMN still has a poor renal prognosis [4, 5]. pMN is caused by subepithelial immune deposits and induces changes in the glomerular basement membrane (GBM). Subepithelial deposits impair podocytes and disrupt the glomerular filtration barrier. Damage to the glomerular capillary wall causes large amounts of protein to leak into the urine [6].

Although various clinical and histological findings have been reported as important renal prognostic factors, the most influential factors have been found to be urinary protein and renal function [1–3]. Patients with pMN have leakage of macromolecules such as IgG due to glomerular damage, and of small molecules such as β2-microglobulin due to tubulointerstitial damage [7–9]. Therefore, disruption of the glomerular capillary wall in membranous nephropathy (MN) may result in the leakage of proteins of various molecular weights.

Fractional excretion of total protein (FETP), which is protein clearance divided by creatinine clearance, has been reported to be a protein leakage indicator that accounts for creatinine kinetics and serum protein levels [10, 11]. There are two important mechanisms for proteinuria. The first is an increase in the permeability of glomerular capillary wall allowing proteins to pass through the glomerulus. The second is a disruption of protein reabsorption mechanism by the epithelial cells of proximal tubules. This is due to an increase in the load of abnormally filtered proteins in the tubular lumen [12]. Unlike proteins, creatinine is excreted in the urine after passing freely through the glomeruli and being secreted in the tubules [13, 14]. FETP is the percentage of filtered proteins excreted in the urine, taking into account creatinine kinetics. In clinical practice, FETP is used as an indicator for pathological assessment in cases of glomerular diseases [15–17]. FETP has been reported to be more valuable than proteinuria as a predictor of renal prognosis in postrenal transplant patients [10]. Patients with pMN have varying degrees of proteinuria, from mild to severe, and some patients present with acute kidney injury. [18, 19]. Since FETP is tightly associated with proteinuria and creatinine kinetics, it may accurately reflect the disruption of glomerular filtration barrier and may be a stronger predictor of renal prognosis in patients with pMN.

Therefore, we aimed to investigate the disruption of the glomerular filtration barrier in Japanese patients with pMN, using FETP, and to examine the association between renal prognosis and FETP.

## MATERIALS AND METHODS

### Participants

This retrospective observational study included patients who were newly diagnosed with MN by kidney biopsy from 2010 to 2022 at Jikei University Hospital, Tokyo, Japan, and its affiliated hospitals (Jikei University Katsushika Medical Center and Jikei University Kashiwa Hospital). The study protocol was approved by the Ethics Review Board of the Jikei University School of Medicine (34-170-11321), and the study followed the tenets of the Declaration of Helsinki. Since this was a retrospective cohort study, information on the research plan was proposed, and an opportunity to opt out was provided; therefore, individual informed consent was not required.

### Exclusion criteria

The exclusion criteria were as follows: age <18 years; unavailable clinical data; fewer than six glomeruli in kidney biopsy specimens; not first-onset pMN; follow-up time <6 months [1]; other diseases treated with prednisolone (PSL) or other immunosuppressants at that time or previously; and presence of secondary factors, including malignant tumors, rheumatic diseases, lupus erythematous, and hepatitis B or C virus infection as confirmed by blood testing, computed tomography and endoscopy [1, 20].

### Definition

Nephrotic syndrome was defined as urinary protein excretion (UPE) of ≥3.5 g/day, with a serum albumin level of ≤3.0 g/dL or a serum total protein level of ≤6.0 g/dL. Complete remission (CR) was defined as urine protein of *<*0.3 g/day. Incomplete remission (ICR) was divided into the following two grades: ICR I (urine protein of *<*1.0 g/day) and ICR II (urine protein of 1.0–3.5 g/day). No response (NR) was defined as the persistence of nephrotic syndrome [21]. Relapse was defined as a rise in proteinuria of >1.0 g/day after CR or ICR I at 6 months [22].

We evaluated the FETP at baseline FETP and at 6 months after biopsy as a predictor of renal prognosis. Urinary protein and glomerular filtration rate during the follow-up period, not just baseline data, have been shown to improve risk prediction in patients with pMN [3, 23, 24]. The Toronto Risk Score includes a combination of renal function and proteinuria parameters. This score was calculated after 12–24 months of follow-up and showed good performance [25]. The Kidney Disease: Improving Global Outcomes (KDIGO) guidelines, which incorporate the results of these previous reports, also use the change in proteinuria after 6 months for risk classification [26].

### Histological analysis

All kidney tissues were obtained percutaneously from all patients during routine renal biopsies. The kidney tissues were embedded in paraffin; cut into 2- to 3-μm sections; and stained with hematoxylin–eosin, periodate–Schiff, Masson trichrome and periodic acid–silver methenamine stain for light microscopy. Formalin-fixed, paraffin-embedded tissue sections of all biopsy samples were subjected to immunohistochemical staining with the routine panel of antibodies, including immunoglobulins (IgG, IgA, IgM) and complement components (C3 and C1q). Biopsy samples fixed with glutaraldehyde were subjected to electron microscopy. We used the pathological findings from tissue samples that were assessed by at least two pathologists. The percentages of all glomeruli in the biopsy samples with global glomerulosclerosis were calculated. The presence of segmental glomerulosclerosis was evaluated. The severity of arteriosclerosis was evaluated using the most severe lesions and was graded as 0 or 1 (Grade 0: intimal thickening < thickness of media; and Grade 1: intimal thickening ≥ media thickness). The degree of tubulointerstitial injury was the sum of the area of interstitial fibrosis/tubular atrophy (IF/TA). IF/TA was graded as follows: Grade 0 (<10%), Grade 1 (10%–25%), Grade 2 (26%–50%) or Grade 3 (>50%) [27]. GBM alterations were classified by electron microscopy according to the classification of Ehrenreich and Churg (EC) (Stage I–IV) [28]. If multiple stages coincided in the same kidney biopsy specimen, the higher stage was selected in the analysis.

### Outcome

**Figure 1:**
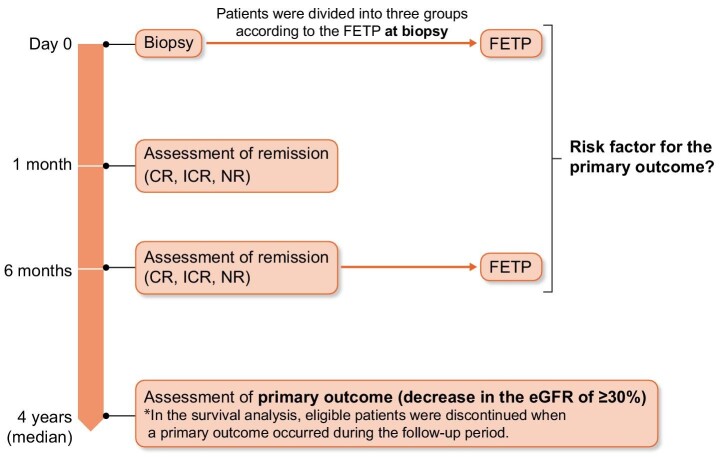
Time course and the definition of primary outcome.

The primary outcome of this study was a decrease in the estimated GFR (eGFR) of ≥30% from the baseline level [29] (Fig. 1). Remission, death and ESKD progression were also examined.

### Measurements

Demographic and laboratory data were obtained from the medical records at the kidney biopsy. The mean arterial pressure was defined as the diastolic blood pressure plus the pulse pressure divided by 3, and hypertension was defined as a systolic blood pressure of >140 mmHg and/or a diastolic blood pressure of >90 mmHg, or the use of antihypertensive medications. The eGFR was calculated using the following formula for Japanese patients: eGFR (mL/min/1.73 m^2^) = 194 × Cr^−1.094^ × age^−0.287^ × (0.739 for women) [30]. FETP was calculated as follows: (serum creatinine × urine protein)/(serum protein × urine creatinine) % [10].

### Statistical analysis

Continuous variables are presented as medians and tertile ranges or numbers with percentages in parentheses. Differences in continuous and categorical variables were evaluated using the Mann–Whitney *U* test and the chi-square test, respectively. The Jonckheere–Terpstra test was used to detect trends in baseline characteristics and morphological measurements according to the FETP tertile. Survival analysis was performed to test the association between renal survival and each parameter collected. Survival time was the time from the first to last follow-up or the time to renal failure. The receiver operating characteristic (ROC) curve analyses were performed for evaluation and comparison of different tests in terms of predictive value using area under the curve (AUC). Univariate comparisons of renal survival were performed using Kaplan–Meier curves and the log-rank test. A Cox regression model was used to investigate the relationship between poor renal outcomes and histopathological or clinical variables. The prognostic factors of renal dysfunction in patients with pMN, namely, age, eGFR, PCR and treatment, were included in the multivariate analysis, and the hazard ratio (HR) was calculated for the risk of developing the primary outcome. Statistical significance was defined as a *P*-value <.05 (two-sided). All statistical analyses were performed using IBM SPSS Statistics for Windows version 29.0 (IBM Corp., Armonk, NY, USA).

## RESULTS

### Baseline characteristics and treatments of patients with pMN

**Figure 2:**
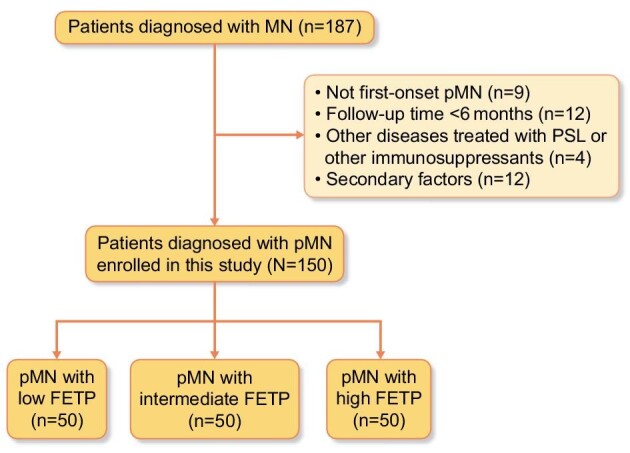
Flow diagram of study participants.

The study identified a total of 187 patients who were diagnosed with MN. Of these 187 patients, 37 were excluded (Fig. 2).

The clinical and laboratory findings of the remaining 150 patients at the time of biopsy diagnosis are shown in Table 1. The median age of the patients at diagnosis was 69.0 (interquartile range 61.0–75.0) years, and 72.7% of the patients were male. The median eGFR at diagnosis was 65.7 (48.3–78.0) mL/min/1.73 m^2^, total protein level was 5.3 (4.6–5.9) g/dL and UPE was 4.0 (1.7–6.3) g/day. According to the tertile of the FETP values at the time of kidney biopsy, patients were divided into three groups. Patients in the high FETP group (0.139%–2.27%) had lower serum albumin levels, higher total cholesterol levels and higher UPE values compared with the findings in the intermediate FETP group and low FETP group (Table 1). The median initial PSL dose was 0.6 (0.5–0.7) mg/kg, and it was not associated with FETP. More patients in the low FETP group than in the high FETP group were on conservative therapy (angiotensin-converting enzyme inhibitors or angiotensin receptor blockers alone) at the time of biopsy diagnosis (*P* < .001). Steroids and additional immunosuppressive therapies were commonly used in the high FETP group. We suspected that patients with higher FETP values have lower remission rates and need intensified immunosuppressive therapy.

**Table 1:** Baseline characteristics at the time of kidney biopsy among all participants and according to the FETP tertile.

Factor	Overall (*N *= 150)	Low FETP group (0.001%–0.052%) (*n* = 50)	Intermediate FETP group (0.053%–0.138%) (*n* = 50)	High FETP group (0.139%–2.27%) (*n* = 50)	*P*-value
Characteristics					
Age (years)	69.0 (61.0–75.0)	66.5 (57.0–71.5)	68.5 (59.0–76.3)	72.0 (65.0–76.3)	.004
Male, *n* (%)	109 (72.7)	33 (66.0)	33 (66.0)	43 (86.0)	.025
BMI (kg/m^2^)	23.7 (21.6–26.0)	23.1 (21.4–25.5)	23.9 (21.4–25.9)	23.7 (21.7–27.2)	.416
MAP (mmHg)	96.0 (88.3–104.0)	96.0 (87.3–101.7)	95.0 (87.5–108.0)	100.0 (90.5–110.0)	.115
Diabetes mellitus, *n* (%)	22 (14.8)	4 (8.0)	4 (8.0)	14 (28.6)	.004
Hypertension, *n* (%)	78 (54.9)	22 (45.8)	23 (52.3)	33 (66.0)	.045
Follow-up period (years)	4.0 (2.0–7.5)	5.0 (3.0–9.0)	3.0 (1.0–6.0)	3.0 (1.0–7.0)	.008
Laboratory data					
TP (g/dL)	5.3 (4.6–5.9)	6.3 (5.8–6.7)	5.3 (4.5–5.5)	4.6 (4.4–5.1)	<.001
Alb (g/dL)	2.3 (1.8–3.0)	3.4 (2.9–3.7)	2.4 (1.9–2.8)	1.6 (1.4–2.0)	<.001
Cr (mg/dL)	0.9 (0.7–1.2)	0.8 (0.6–0.9)	0.7 (0.6–1.0)	1.2 (0.9–1.4)	<.001
eGFR (mL/min/1.73 m^2^)	65.7 (48.3–78.0)	74.6 (64.8–85.9)	68.4 (57.6–84.5)	47.1 (37.0–61.7)	<.001
IgG (mg/dL)	845.5 (578.8–1070.8)	980.0 (860.5–1179.5)	709.0 (526.0–947.0)	672.5 (531.3–967.8)	<.001
IgA (mg/dL)	246.0 (196.0–332.5)	248.0 (186.8–312.8)	237.0 (171.5–333.5)	256.0 (209.0–335.0)	.588
IgM (mg/dL)	84.0 (64.0–125.0)	88.0 (61.5–117.8)	78.0 (64.5–118.0)	90.0 (65.0–141.5)	.544
C3 (mg/dL)	114.0 (97.0–129.0)	111.0 (95.0–125.0)	112.0 (94.0–126.0)	124.0 (110.5–134.3)	.009
C4 (mg/dL)	30.0 (24.3–34.0)	25.0 (21.0–31.0)	30.0 (25.0–33.0)	32.0 (29.0–39.3)	<.001
T-Cho (mg/dL)	253.5 (197.2–331.6)	211.9 (173.1–255.5)	260.3 (211.4–352.0)	297.0 (249.0–393.2)	<.001
HbA1c (%)	5.6 (5.4–5.9)	5.6 (5.4–5.8)	5.6 (5.4–5.9)	5.7 (5.5–6.3)	.027
UPE (g/day)	4.0 (1.7–6.3)	1.0 (0.6–2.2)	4.2 (3.1–5.8)	6.4 (4.3–9.3)	<.001
PCR (g/gCr)	5.3 (2.6–8.0)	1.4 (0.8–2.7)	5.3 (4.3–6.9)	9.1 (7.5–11.0)	<.001
FETP (%)	0.08 (0.03–0.16)	0.02 (0.01–0.03)	0.08 (0.07–0.11)	0.20 (0.16–0.30)	<.001
Treatment					
Only ACEi or ARB therapy, *n* (%)	56 (37.3)	35 (70.0)	11 (22.0)	10 (20.0)	<.001
PSL, *n* (%)	49 (32.7)	8 (16.0)	23 (46.0)	18 (36.0)	.034
PSL + other immunosuppressants, *n* (%)	45 (30.0)	7 (14.0)	16 (32.0)	22 (44.0)	.001
PSL + CyA, *n* (%)	43 (28.7)	7 (17.1)	16 (39.0)	18 (43.9)	
PSL + Tac, *n* (%)	4 (2.7)	0 (0.0)	0 (0.0)	4 (100.)	
PSL + MZB, *n* (%)	1 (0.7)	0 (0.0)	0 (0.0)	1 (100.0)	
PSL + CPA, *n* (%)	1 (0.7)	0 (0.0)	0 (0.0)	1 (100.0)	
Dose of initial PSL (mg)	40 (30–40)	32.5 (30–40)	40 (40–40)	40 (30–40)	.269
Dose of initial PSL (mg/kg)	0.6 (0.5–0.7)	0.49 (0.43–0.70)	0.59 (0.56–0.69)	0.62 (0.53–0.68)	.230
Outcome					
Outcome at 1 month					
CR, *n* (%)	4 (2.7)	3 (6.1)	0 (0.0)	1 (2.0)	.211
ICR Ⅰ, *n* (%)	25 (16.8)	16 (32.7)	5 (10.0)	4 (8.0)	.001
ICR Ⅱ, *n* (%)	59 (39.6)	23 (46.9)	20 (40.0)	16 (32.0)	.130
No response, *n* (%)	61 (40.9)	7 (14.3)	25 (50.0)	29 (58.0)	<.001
Outcome at 6 months					
CR, *n* (%)	29 (20.1)	9 (18.4)	9 (18.4)	11 (23.9)	.509
ICR Ⅰ, *n* (%)	41 (28.5)	20 (40.8)	11 (22.4)	10 (21.7)	.037
ICR Ⅱ, *n* (%)	44 (30.6)	17 (34.7)	17 (34.7)	10 (21.7)	.179
No response, *n* (%)	30 (20.8)	12 (24.5)	15 (32.6)	15 (32.6)	.001
Outcome at last follow-up					
Decrease in eGFR >30%, *n* (%)	38 (25.3)	6 (12.0)	12 (24.0)	20 (40.0)	.001
Day to decrease in eGFR >30% (years)	2.0 (0.0–4.3)	5.0 (3.3–6.5)	1.5 (0.3–3.0)	1.5 (0.0–3.8)	.061
Relapse, *n* (%)	36 (51.4)	15 (51.7)	11 (55.0)	10 (47.6)	.817
ESKD, *n* (%)	5 (3.3)	0 (0.0)	1 (2.0)	4 (8.0)	.026
Death, *n* (%)	6 (4.0)	0 (0.0)	1 (2.0)	5 (10.0)	.011

Values are presented as median (interquartile range) or *n* (%).

Conversion factors for units: serum total cholesterol in mg/dL to mmol/L, ×0.02586; Cr in mg/dL to µmol/L, ×88.4.

There were a total of 12 missing values.

BMI, body mass index; MAP, mean arterial pressure; TP, total protein; Alb, albumin; Cr, creatinine; IgG, immunoglobulin G; IgA, immunoglobulin A; IgM, immunoglobulin M; C3, complement 3; C4, complement 4; T-Cho, total cholesterol; ACEi, angiotensin-converting enzyme inhibitor; ARB, angiotensin receptor blocker.

The primary outcome was observed in 38 patients (25.3%), and the frequency of the primary outcome was greater in the high FETP group than in the low FETP group (*P* = .001). The number of years for the eGFR to decrease by >30% was not significantly different among the three groups. At the 1-month and 6-month time points, more patients had NR in the high FETP group than in low FETP group. Death and ESKD were noted in five and six patients, respectively, in the overall study population, and these outcomes were more frequent in the high FETP group than in the low FETP group. Relapse after CR or ICR at 6 months was not associated with FETP.

Supplementary data, Table S1 presents a comparison between the primary outcome incidence group and the primary outcome non-incidence group. The FETP values at baseline and at 6 months were higher in the primary outcome incidence group. Although incidences of diabetes mellitus and hypertension were higher in the high FETP group, these were not significantly different compared with those in the groups with or without the primary outcome.

Table 2 presents the relationship between histological findings and FETP in patients with pMN. In EC stage classification, FETP tended to be high in Stage Ⅱ and low in Stages Ⅲ and Ⅳ. There was no significant difference in Stage Ⅰ. Global glomerulosclerosis and IF/TA were more frequent in the high FETP group than in the low FETP group.

**Table 2:** Pathological findings at the time of kidney biopsy among all participants and according to the FETP tertile.

Factor	Overall (*N *= 150)	Low FETP group (0.001%–0.052%) (*n* = 50)	Intermediate FETP group (0.053%–0.138%) (*n* = 50)	High FETP group (0.139%–2.27%) (*n* = 50)	*P*-value
EC classification					
Stage I	33 (22.0)	6 (12.0)	13 (26.0)	14 (28.0)	.054
Stage II	72 (48.0)	19 (38.0)	23 (46.0)	30 (60.0)	.028
Stage III	31 (20.7)	17 (34.0)	10 (20.0)	4 (8.0)	.001
Stage IV	14 (9.3)	8 (16.0)	4 (8.0)	2 (4.0)	.040
Global glomerulosclerosis, *n* (%)	10.0 (2.5–16.7)	5.3 (0.0–15.0)	8.3 (0.0–15.5)	13.8 (6.6–19.4)	.003
Segmental sclerosis, *n* (%)	22 (14.7)	7 (14)	5 (10)	10 (20)	.398
Interstitial fibrosis/tubular atrophy					
Grade 0	50 (37.0)	25 (52.1)	16 (37.2)	9 (20.5)	.002
Grade 1	74 (54.8)	22 (45.8)	24 (55.8)	28 (63.6)	.087
Grade 2	10 (7.4)	1 (2.1)	3 (7.0)	6 (13.6)	.036
Grade 3	1 (0.7)	0 (0.0)	0 (0.0)	1 (2.3)	.215
Arteriosclerotic lesions, *n* (%)					
Grade 0	73 (52.9)	25 (55.6)	25 (52.1)	23 (51.1)	.674
Grade 1	65 (47.1)	20 (44.4)	23 (47.9)	22 (48.9)	

Values are presented as median (interquartile range) or *n* (%).

There were a total of 15 missing values.

ROC curves were constructed to evaluate and compare the different tests (Supplementary data, Fig. S1a and b). The AUCs of the baseline and 6-month FETPs for predicting the primary outcome were 0.67 and 0.70, respectively (Supplementary data, Figures, Table S2a and b).

### Kaplan–Meier curves

**Figure 3:**
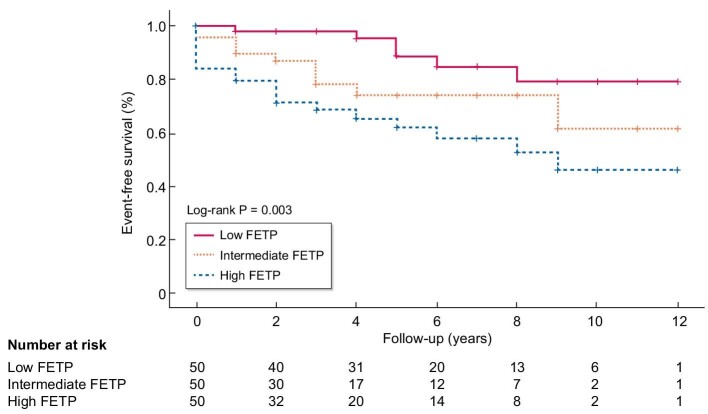
Kaplan–Meier plots of event-free survival stratified by FETP.

Kaplan–Meier survival analysis was performed to assess the primary outcome according to the three FETP groups. The incidence of the primary outcome was significantly higher in the high FETP group than in the low and intermediate FETP groups (log-rank test, *P* = .003) (Fig. 3).

### Univariate and multivariate Cox proportional hazard analyses of the primary outcome

Tables 3a and b presents the results of the univariate and multivariate regression analyses using the Cox proportional hazards model. In the univariate analysis, the primary outcome was significantly associated with FETP, age, PCR and treatment. In the multivariate analysis adjusted for age, eGFR, PCR and treatment, FETP at the kidney biopsy was significantly associated with an eGFR decrease of ≤30% (adjusted HR 8.19; 95% confidence interval 1.41–47.62; *P* = .019) (Table 3a). FETP at 6 months was also significantly associated with the primary outcome in the multivariate analysis (Table 3b). There was no multicollinearity between FETP and PCR. Furthermore, multivariate analysis including diabetes mellitus and hypertension as covariates revealed that FETP value at the time of kidney biopsy and 6 months remained a risk factor for the primary outcome (Supplementary data, Tables S3a and b).

**Table 3a:** Univariate and multivariate Cox proportional hazard analyses using data at kidney biopsy for the primary outcome.

	Unadjusted			Multivariate model		
Variable	HR	95% CI	*P-*value	HR	95% CI	*P-*value
FETP at kidney biopsy	5.05	2.46–10.34	<.001	8.19	1.41–47.62	0.019
Age	1.06	1.02–1.10	.002	1.08	1.04–1.13	<0.001
eGFR at kidney biopsy	0.99	0.97–1.00	.076	1.02	0.99–1.04	0.124
PCR at kidney biopsy	1.10	1.05−1.16	<.001	0.99	0.89−1.09	0.785
Treatment						
PSL^a^	1.13	0.48–2.67	.779	0.99	0.41–2.42	.996
PSL + other immunosuppressants^a^	1.84	0.84–4.01	0.125	2.39	1.01–5.649	.047

The multivariable model was adjusted for age, eGFR, PCR and treatment.

^a^Angiotensin-converting enzyme inhibitor or angiotensin receptor blocker therapy was used as the reference.

CI, confidence interval.

**Table 3b:** Univariate and multivariate Cox proportional hazard analyses using data at 6 months for the primary outcome.

	Unadjusted			Multivariate model		
Variable	HR	95% CI	*P-*value	HR	95% CI	*P-*value
FETP at 6 months	8.44	2.91–24.47	<.001	17.8	2.06–154.5	.009
Age	1.06	1.02–1.10	.002	1.08	1.02–1.15	.007
eGFR at 6 months	0.96	0.94–0.98	<.001	0.99	0.96–1.02	.435
PCR at 6 months	1.13	1.04–1.22	.004	0.93	0.81–1.06	.280
Treatment						
PSL^a^	1.13	0.48–2.67	.779	0.78	0.31–1.98	.599
PSL + other immunosuppressants^a^	1.84	0.84–4.01	.125	2.35	0.99–5.55	.051

The multivariate model was adjusted for age, eGFR, PCR and treatment.

^a^Angiotensin-converting enzyme inhibitor or angiotensin receptor blocker therapy was used as the reference.

CI, confidence interval.

## DISCUSSION

There were two main novel findings in this study. First, FETP was correlated with the clinicopathological severity of pMN. Second, FETP was more useful than PCR for renal prognostic factor in pMN. FETP at the time of kidney biopsy was associated with NR an eGFR decrease of ≥30%, and progression to ESKD and death. Moreover, FETP at baseline and at the time of therapeutic evaluation were critical renal prognostic factors.

In a normal kidney, proteins filtered by the glomerulus are not detected in urine because they are reabsorbed by megalin–tubulin complex in the proximal tubules [31, 32]. In pMN, glomerular urinary protein is excreted in excess of the reabsorptive capacity in the tubules, or tubular urinary protein is excreted with reduced reabsorptive capacity owing to tubular damage [11]. Even when excreting the same urinary protein, the degree to which the serum protein is maintained by hepatic synthesis varies among individuals [33–35]. Therefore, FETP may be an index that can more accurately reflect the protein kinetics of pMN through consideration of protein clearance and creatinine clearance. Furthermore, FETP may be an accurate indicator of pMN severity. The results revealed that the high FETP group had more severe proteinuria and hypoalbuminemia as well as lower IgG levels. Additionally, hypoalbuminemia induced hepatic resynthesis of protein, resulting in elevated levels of cholesterol, C3 and C4.

FETP was associated with clinicopathological findings. Patients with increased FETP had a higher frequency of global glomerulosclerosis in this study. Sun *et al*. have reported that a high level of glomerulosclerosis was an independent risk factor for the prognosis of patients with pMN [20]. The deposition of subepithelial immune complexes in the glomerulus can affect podocyte and basement membrane attachment, and the detachment of epithelial cells and the GBM can cause glomerulosclerosis [36]. Since FETP is correlated with the severity of pMN, the finding of this study that it was associated with glomerulosclerosis is consistent with the findings of previous reports. Additionally, FETP was associated with EC stage classification. FETP tended to be high in Stage Ⅱ, and conversely, it tended to be low in stages Ⅲ and Ⅳ.

Although the effect of EC stage as a predictor of renal prognosis in patients with pMN remains controversial, some studies suggested that EC stage was associated with a clinical stage in patients with MN. While Marx *et al*. presented an advanced EC stage as a poor renal prognostic factor in patients with MN [37], Shiiki *et al*. showed that EC stage was not associated with renal prognosis [1]. Ehrenreich *et al*. explained that patients with MN showed restoration of a normal GBM at the time of CR, defined as Stage IV [28]. Based on the results of this study, FETP was suggested to be associated with the activity and recovery period in pMN from a histological point of view.

The ability of FETP to predict the risk of renal failure remained unchanged when known risk factors, such as age, PCR, eGFR and treatment, were considered in the multivariate analysis. Clinical factors, such as older age [1, 2, 38], male sex [1], serum creatinine levels [1, 2, 3], the severity of proteinuria [2, 38, 39] and hypertension [39], and histological factors, such as the degree of tubulointerstitial damage [39] and glomerulosclerosis [20], have been reported as risk factors for renal prognosis in patients with pMN. Among all the predictive variables reported in previous studies, the most frequently used and reliable indicators were proteinuria and renal function variables [2]. Therefore, the KDIGO 2021 guidelines recommend treating pMN according to a risk classification that includes proteinuria and eGFR [26]. Although this classification is valid, the problem remains that most patients’ disease characteristics do not fit perfectly into one category, and the risk classification needs to be more accurate [6]. Based on the results of this study, FETP has the potential to simplify this complex classification and solve the problem. Additionally, this study suggested the usefulness of FETP in follow-up, making it a handy indicator for predicting renal prognosis.

The treatment selection regarding pMN also affects remission and renal prognosis. Immunosuppressants were significantly more effective than conservative treatments in terms of CR and PR [6, 40]. Therefore, treatment content was included in the multivariate analysis. We found that FETP was associated with renal prognosis at baseline and at the time of treatment decision, regardless of the treatment.

This study has several limitations. First, we could not measure autoantibodies to the M-type phospholipase A2 receptor (PLA2R) and stain for PLA2R. However, since we routinely search for malignancies and infections causing secondary MN at the time of diagnosis, we could adequately exclude secondary MN. Second, all the study patients were Japanese. The outcome of pMN appears to be affected by geography and race. pMN is thought to have a more benign course in Japanese people than in Caucasians [1, 41]. Therefore, FETP in other racial groups should be considered. Despite these limitations, FETP was found to be a valuable indicator for predicting renal prognosis in patients with pMN.

In conclusion, this study revealed that FETP reflected the pathophysiology in pMN, and patients with a high FETP value had a poor renal prognosis. FETP at baseline and at the time of therapeutic evaluation were found to be more important renal prognostic factors than PCR. FETP can easily be calculated from only serum and urine examinations, which are routine laboratory examinations. Thus, FETP should be considered for use in the prediction of renal outcomes in Japanese patients with pMN.

## Supplementary Material

sfae071_Supplemental_Files

## Data Availability

The datasets used and/or analyzed during the current study are available from the corresponding author upon reasonable request.
