# Supplementary material for: Fractional excretion of total protein predicts renal prognosis in Japanese patients with primary membranous nephropathy
Source: Clin Kidney J. 2024 Mar 20;17(5):sfae071. doi: 10.1093/ckj/sfae071 (PMC11063954; doi:10.1093/ckj/sfae071)

Supplementary Figure 1a. Receiver operator characteristic curve analysis for predicting relapse using FETP and PCR at kidney biopsy

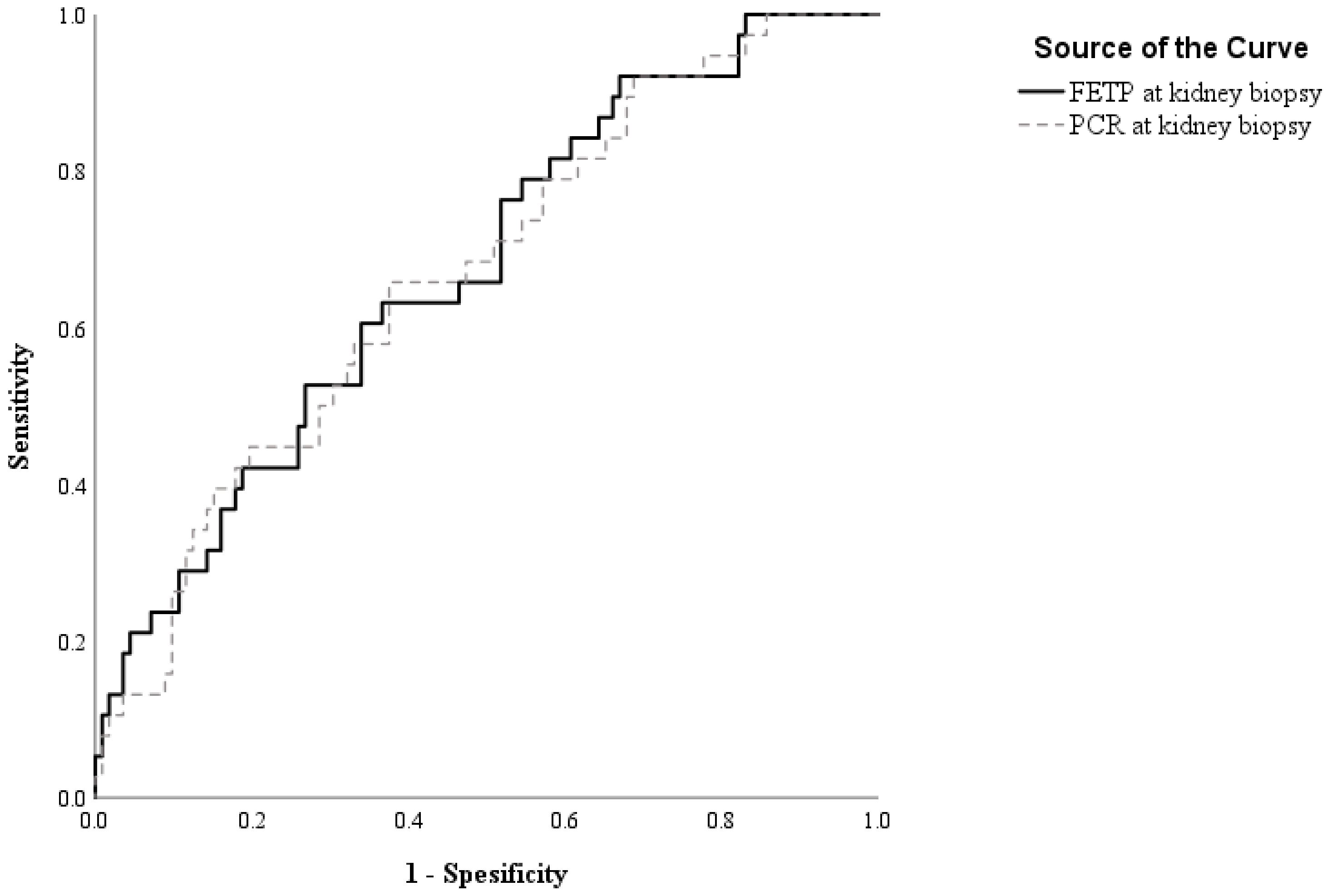

Supplementary Figure 1b. Receiver operator characteristic curve analysis for predicting relapse using FETP and PCR at 6 months

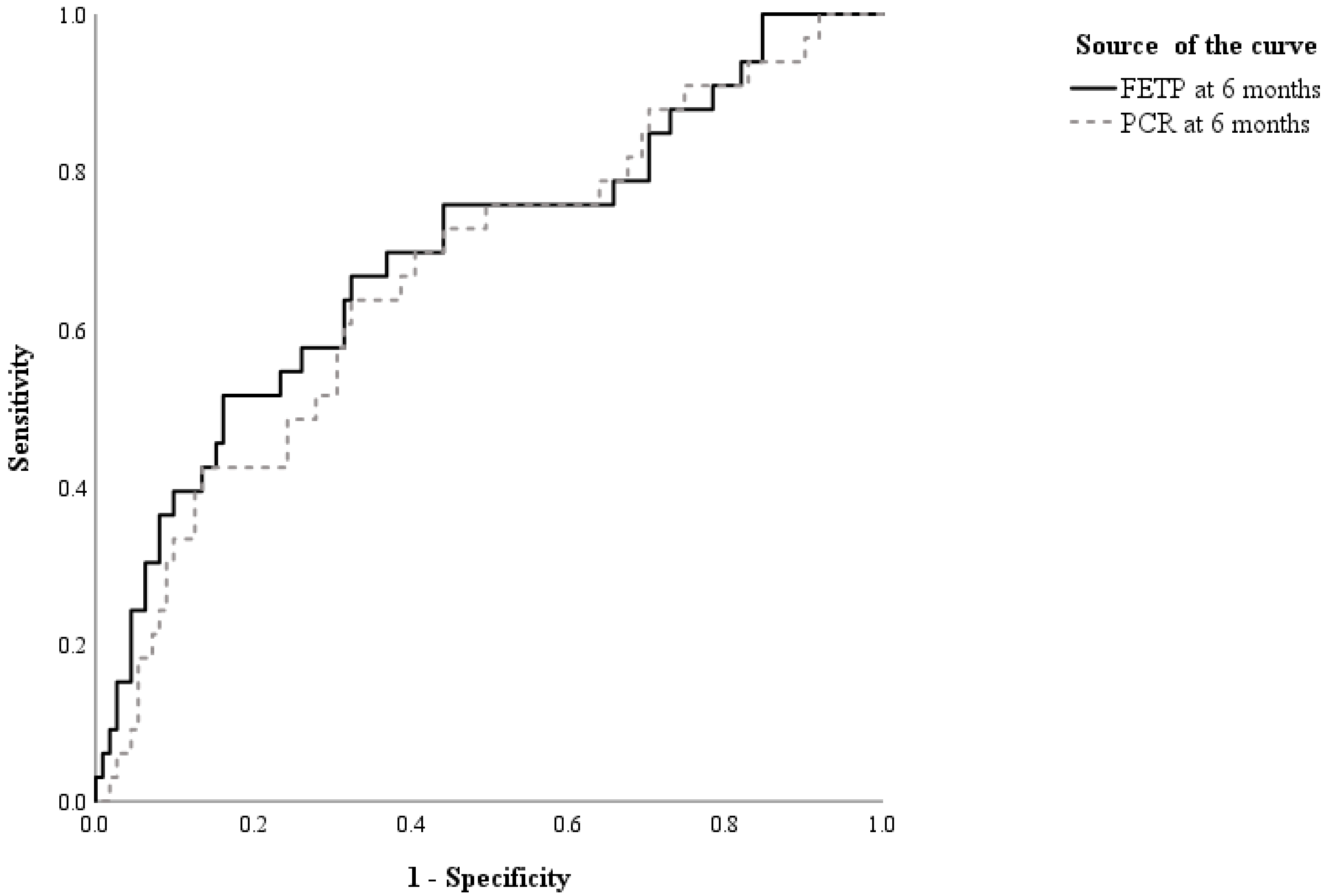

Supplement: sfae071_Supplemental_Files [file sfae071_supplemental_files.zip › Supplementary_Figure1_ROC_CKJ_3rd submit.pdf]
