# Supplementary material for: Fractional excretion of total protein predicts renal prognosis in Japanese patients with primary membranous nephropathy
Source: Clin Kidney J. 2024 Mar 20;17(5):sfae071. doi: 10.1093/ckj/sfae071 (PMC11063954; doi:10.1093/ckj/sfae071)
Supplement: sfae071_Supplemental_Files [file sfae071_supplemental_files.zip › Supplementary_Table1_CKJ_proof submit.pdf]

Supplementary Table 1. Baseline characteristics at the time of kidney biopsy among all participants and according to the presence or absence of the primary outcome

Factor	Overall<br>$N = 150$	Primary outcome non-incidence group (n = 112)	Primary outcome incidence group (n = 38)	P value
**Characteristics**				
Age (years)	69.0 (61.0–75.0)	68.0 (59.0–74.0)	72.0 (66.8–80.3)	0.004
Male, n (%)	109 (72.7)	78 (69.6)	31 (81.6)	0.154
BMI (kg/m^2)	23.7 (21.6–26.0)	23.3 (21.4–25.8)	24.2 (22.0–27.0)	0.209
MAP (mmHg)	96.0 (88.3–104.0)	94.3 (86.7–102.7)	102.7 (93.0–112.0)	0.004
Diabetes mellitus, n (%)	22 (14.8)	13 (11.7)	9 (23.7)	0.073
Hypertension, n (%)	78 (54.9)	52 (50.0)	26 (68.4)	0.058
Nephrotic syndrome, n (%)	103 (68.7%)	70 (62.5)	33 (86.6)	0.003
AKI, n (%)	23 (15.3)	16 (20.3)	7 (25.0)	0.601
Follow-up period (years)	4.0 (2.0–7.5)	4.0 (2.0–8.0)	3.4 (2.0–0.8)	0.622
**Laboratory data**				
TP (g/dL)	5.3 (4.6–5.9)	5.4 (4.6–6.2)	4.9 (4.2–5.4)	0.003
Alb (g/dL)	2.3 (1.8–3.0)	2.6 (1.9–3.3)	1.9 (1.5–2.5)	0.001
Cr (mg/dL)	0.9 (0.7–1.2)	0.8 (0.7–1.1)	0.9 (0.8–1.3)	0.137
eGFR (mL/min/1.73 m^2)	65.7 (48.3–78.0)	66.7 (53.0–78.8)	58.6 (42.2–74.9)	0.143
IgG (mg/dL)	845.5 (578.8–1070.8)	854.0 (594.0–1075.0)	781.0 (556.5–1107.0)	0.626
IgA (mg/dL)	246.0 (196.0–332.5)	234.0 (186.3–315.8)	299.0 (207.5–379.8)	0.067
IgM (mg/dL)	84.0 (64.0–125.0)	84.0 (66.0–123.0)	91.5 (53.3–133.5)	0.996
C3 (mg/dL)	114.0 (97.0–129.0)	112.0 (95.0–128.0)	123.0 (109.0–132.0)	0.108
C4 (mg/dL)	30.0 (24.3–34.0)	29.0 (24.0–34.0)	32.0 (27.5–38.0)	0.043
T-Cho (mg/dL)	253.5 (197.2–331.6)	250.5 (193.3–333.6)	260.5 (204.5–332.3)	0.619
HbA1c (%)	5.6 (5.4–5.9)	5.6 (5.4–5.9)	5.7 (5.5–6.2)	0.208
PCR (g/gCr)	5.3 (2.6-8.0)	4.6 (1.9–7.6)	7.1 (3.9–10.3)	0.002
UPE (g/day)	4.0 (1.7–6.3)	3.5 (1.0–6.0)	5.0 (3.2–7.4)	0.004
FETP (%)	0.08 (0.03–0.16)	0.08 (0.03–0.14)	0.14 (0.07–0.22)	0.001
**Treatment**				

Only ACEi or ARB therapy, n (%)	56 (37.3)	43 (38.7)	11 (29.7)	0.431
PSL, n (%)	49 (32.7)	39 (35.1)	10 (27.0)	0.424
PSL+ other immunosuppressants, n (%)	45 (30.0)	29 (26.1)	16 (43.2)	0.042
Dose of initial PSL (mg)	40 (30–40)	40.0 (30–40)	40 (40–40)	0.555
Dose of initial PSL (mg/kg)	0.6 (0.5–0.7)	0.60 (0.54–0.69)	0.60 (0.55–0.68)	0.895
**Follow-up at 6 months**				
TP (g/dL)	6.0 (5.4–6.6)	6.0 (5.6–6.6)	5.8 (5.2–6.1)	0.008
Cr (mg/dL)	0.9 (0.8–1.1)	0.9 (0.8–1.1)	1.1 (0.8–1.7)	0.002
eGFR (mL/min/1.73 m^2)	61.9 (46.3–76.5)	64.0 (52.0–77.0)	48.3 (27.4–72.5)	0.002
PCR (g/gCr)	1.1 (0.4–2.8)	0.9 (0.3–2.2)	2.1 (0.7–5.8)	0.003
FETP (%)	0.01 (0.01–0.05)	0.01 (0.00–0.04)	0.05 (0.01–0.13)	<0.001

Note: Values are presented as median (interquartile range) or n (%).

Conversion factors for units: serum total cholesterol in mg/dL to mmol/L, ×0.02586; Cr in mg/dL to μmol/L, ×88.4.

Abbreviations: AKI, acute kidney injury; BMI, body mass index; MAP, mean arterial pressure; TP, total protein; Alb, albumin; Cr, creatinine; eGFR, estimated glomerular filtration rate; IgG, immunoglobulin G; IgA, immunoglobulin A; IgM, immunoglobulin M; C3, complement 3; C4, complement 4; T-Cho, total cholesterol; UPE, urinary protein excretion; FETP, fractional excretion of total protein; ACEi, angiotensin-converting enzyme inhibitor; ARB, angiotensin receptor blocker; PSL, prednisolone; PCR, protein–creatinine ratio

There were a total of 12 missing values.
