# Supplementary material for: Fractional excretion of total protein predicts renal prognosis in Japanese patients with primary membranous nephropathy
Source: Clin Kidney J. 2024 Mar 20;17(5):sfae071. doi: 10.1093/ckj/sfae071 (PMC11063954; doi:10.1093/ckj/sfae071)
Supplement: sfae071_Supplemental_Files [file sfae071_supplemental_files.zip › Supplementary_Table2_ROC_CKJ_3rd submit.pdf]

Supplementary Table 2a. Receiver Operating Characteristic curve analysis using data at kidney biopsy for predicting for primary outcome

	AUC	Cut-off	Sensitivity (%)	Specificity (%)
FETP at biopsy	0.67	0.10	63.2	63.4
PCR at biopsy	0.67	6.1	65.8	62.5

Note: AUC, area under the curve; Cut off = level above which a test result was deemed significant

Abbreviations: FETP, fractional excretion of total protein; PCR, protein-creatinine ratio;

Supplementary Table 2b. Receiver Operating Characteristic curve analysis using data at 6 months for predicting for primary outcome

	AUC	Cut-off	Sensitivity (%)	Specificity (%)
FETP at 6 months	0.70	0.05	51.5	83.8
PCR at 6 months	0.67	1.5	63.6	67.6

Note: AUC, area under the curve; Cut off = level above which a test result was deemed significant

Abbreviations: FETP, fractional excretion of total protein; PCR, protein-creatinine ratio;
