# Supplementary material for: Fractional excretion of total protein predicts renal prognosis in Japanese patients with primary membranous nephropathy
Source: Clin Kidney J. 2024 Mar 20;17(5):sfae071. doi: 10.1093/ckj/sfae071 (PMC11063954; doi:10.1093/ckj/sfae071)
Supplement: sfae071_Supplemental_Files [file sfae071_supplemental_files.zip › Supplementary_Table3_Cox_CKJ_3rdsubmit.pdf]

Supplementary Table 3a. Univariate and multivariate Cox proportional hazard analyses using data at kidney biopsy for the primary outcome

Variable	Unadjusted			Multivariate model		
	HR	95% CI	*P* value	HR	95% CI	*P* value
FETP at kidney biopsy	5.05	2.46–10.34	<0.001	9.098	1.49-55.69	0.017
Age	1.06	1.02–1.10	0.002	1.083	1.04-1.13	<0.001
eGFR at kidney biopsy	0.99	0.97–1.00	0.076	1.020	1.00-1.04	0.055
PCR at kidney biopsy	1.10	1.05−1.16	<0.001	0.981	0.88-1.09	0.724
Treatment PSL*	1.13	0.48–2.67	0.779	1.044	0.43-2.51	0.923
Treatment PSL + other immunosuppressants*	1.84	0.84–4.01	0.125	2.506	1.06-5.95	0.037
Hypertension	1.89	0.95-3.73	0.070	1.087	0.52-2.29	0.826
Diabetes mellitus	1.89	0.90-4.00	0.095	1.385	0.59-3.23	0.451

Note: The multivariable model was adjusted for age, eGFR, and treatment.

Abbreviations: HR, hazard ratio; CI, confidence interval; FETP, fractional excretion of total protein; eGFR, estimated glomerular filtration rate; PSL, prednisolone

*Angiotensin-converting enzyme inhibitor or angiotensin receptor blocker therapy was used as the reference.

Supplementary Table 3b. Univariate and multivariate Cox proportional hazard analyses using data at 6 months for the primary outcome

Variable	Unadjusted			Multivariate model		
	HR	95% CI	*P* value	HR	95% CI	*P* value
FETP at 6 months	8.44	2.91–24.47	<0.001	8.46	1.10-59.56	0.032
Age	1.06	1.02–1.10	0.002	1.08	1.02-1.14	0.010
eGFR at 6 months	0.99	0.97–1.00	0.076	0.99	0.97-1.02	0.486
PCR at 6 months	1.10	1.05−1.16	<0.001	0.95	0.83-1.09	0.458
Treatment PSL*	1.13	0.48–2.67	0.779	0.84	033-2.15	0.719
Treatment PSL + other immunosuppressants*	1.84	0.84–4.01	0.125	2.54	1.05-6.14	0038
Hypertension	1.89	0.95-3.73	0.070	1.09	0.48-2.44	0.853
Diabetes mellitus	1.89	0.90-4.00	0.095	0.88	0.35-2.23	0.791

Note: The multivariable model was adjusted for age, eGFR, and treatment.

Abbreviations: HR, hazard ratio; CI, confidence interval; FETP, fractional excretion of total protein; eGFR, estimated glomerular filtration rate; PSL, prednisolone

*Angiotensin-converting enzyme inhibitor or angiotensin receptor blocker therapy was used as the reference.
